# A Multi-Layer Multi-Pass Weld Bead Cross-Section Morphology Extraction Method Based on Row–Column Grayscale Segmentation

**DOI:** 10.3390/ma17194683

**Published:** 2024-09-24

**Authors:** Ting Lei, Shixiang Gong, Chaoqun Wu

**Affiliations:** 1 School of Mechanical and Electrical Engineering, Wuhan University of Technology, Wuhan 430070, China; leiting0621@whut.edu.cn (T.L.); gsx1371470161@whut.edu.cn (S.G.); 2 Hubei Provincial Engineering Research Center of Robotics and Intelligent Manufacturing, Wuhan 430070, China

**Keywords:** weld bead morphology, image identification, multi-layer multi-pass, feature extraction method

## Abstract

In the field of welding detection, weld bead cross-section morphology serves as a crucial indicator for analyzing welding quality. However, the extraction of weld bead cross-section morphology often relies on manual extraction based on human expertise, which can be limited in consistency and operational efficiency. To address this issue, this paper proposes a multi-layer multi-pass weld bead cross-section morphology extraction method based on row–column grayscale segmentation. The weld bead cross-section morphology image is pre-processed and then segmented into rows and columns based on the average gray value of the image. In order to extract the feature of multi-layer multi-pass weld feature images, an outline showing the binarization threshold is selected for each segmented image (ESI). Then, the weld contour of ESI is extracted before image fusion and morphological processing. Finally, the weld feature parameters (circumference, area, etc.) are extracted from the obtained weld feature image. The results indicate that the relative errors in circumference and area are within 10%, while the discrepancies in maximum weld seam width and maximum weld seam height can be close to the true value. The quality assessment falls within a reasonable range, the average value of SSIM is above 0.9 and the average value of PSNR is above 60 on average. The results demonstrate that this method is feasible for extracting the general contour features of multi-layer multi-pass weld bead cross-section morphology images, providing a basis for further detailed analysis and improvement in welding quality assessment.

## 1. Introduction

In the current context of continuously increasing demands for product quality and safety, welding technology, as a well-established material thermal processing technique, has consistently been applied across various manufacturing industries such as vehicles, ships, bridges, and aerospace. Simultaneously, in products featuring welded joint structures, the quality of welds plays a decisive role in determining the final product’s quality and safety performance. For instance, in the process of battery production, there is a critical demand for high-quality welding inspection [1].

The quality of weld seam formation is a crucial criterion for evaluating weld quality. A well-formed weld bead should exhibit a surface with uniform, delicate, and aesthetically pleasing ripples. It should also possess correct geometric shapes, appropriate weld seam height, smooth transition between the weld seam and the base material and be devoid of important defects such as undercut and lack of fusion. Post-weld inspection of the weld appearance is an effective method to ensure and enhance the quality of weld seam formation.

Recently, welding methods have become increasingly diverse, resulting in varying characteristics of weld seams. For instance, welding dissimilar materials can lead to asymmetrical features [2]. Laser welding offers high operability but tends to exhibit keyhole features [3]. Multi-layer and multi-pass welding can also result in certain angular distortions [4]. Different steels require different welding methods, and the weldability of steel decreases as the carbon content increases [5]. When the welding speed is increased, there will be some new problems such as undercut and hump, which differ from those of traditional welding speeds [6].

Therefore, when conducting quality inspection of welds, the cross-sectional morphology of the weld will also be an important indicator for evaluating the weld. Currently, the traditional method of appearance inspection involves manual observation by inspectors and simple measurements using inspection tools. This approach often results in substantial workload and lacks rigorous scientific conclusions due to variations in experience and qualifications among inspectors. When faced with hundreds or thousands of inspections, the process is inefficient and has limitations in consistency. Moreover, it primarily provides qualitative detection of defects, lacking a quantitative assessment. In the contemporary context of highly automated welding production, there is a need for a feasible visual automated system for detecting weld morphology. However, traditional ultrasound imaging systems incur high costs in image acquisition [7].

To overcome the reliance on traditional quality inspection methods, which are dependent on experience, have low accuracy, and require long identification times [8], in the contemporary context of highly automated welding production, there is a need for an automated visual inspection system.

Siva Shanmugam et al. [9] used finite element research to carry out three-dimensional finite element modeling of weld morphology and predict parameters. However, such methods require high fitting accuracy of formulas and cannot fully express small feature gaps of welds. Zhao et al. [10] utilized computational fluid dynamics (CFD) to predict weld bead cross-sectional features, but this approach is limited to single-pass welds and cannot accurately depict actual weld cross-section morphology.

Wójcicka et al. [11] identified weld seams by image histogram equalization, filtering, and adaptive binarization and segmentation of the image, but it has more limitations. Ai et al. [12] used segmentation of weld seams using a seed region growing method with automatic selection of initial seeds, which can better extract the weld morphology, but the examined weld features are not complete enough. Zhang et al. [13] adopted adaptive image fusion technology to detect internal features of weld seams, thereby enabling the adaptive extension of the dynamic range of vision detection systems at a relatively low cost. Ting Lei et al. [14] proposed a dynamic threshold adjustment method, which can combine the quick positioning of the Otsu method and the weight balance of the average method which plays a proactive role in monitoring the morphology of small holes in laser welding.

Sun Jiahao et al. [15] employed a neural network to predict the weld bead cross-section morphology of 316L stainless steel laser welding, describing the weld seam contour using Hermite interpolation and least squares fitting. However, the coordinates of the weld seam contour points were obtained from measurement software MATLAB R2019a, introducing inaccuracies in the coordinates. The detection of welding seam images was conducted by Alaknanda et al. [16] using a morphological watershed algorithm, which addressed the issue of over-segmentation in single-layer watershed segmentation. However, the accuracy of extracting the morphology contour of the welding seam was still insufficient.

Therefore, this paper, with the identification and detection of weld seam cross-section morphology as the starting point, proposes a method for processing weld seam cross-section morphology images based on row–column grayscale segmentation. This method enables the automated processing and contour extraction of collected weld cross-section morphology images, obtaining weld characteristic parameters. This represents a new application of image processing technology in the field of weld identification, potentially advancing more precise contour extraction of welds and the prediction of welding process parameters.

## 2. Materials and Methods

### 2.1. Materials Preparation

**Figure 1:**
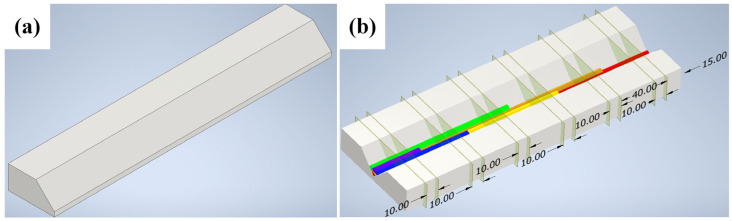
Schematic diagram of material preparation. (**a**) Base material. (**b**) Schematic of welding and EDM.

In this study, the raw materials used were 240 mm × 80 mm × 20 mm thick Q235 steel (Wuhan Hezeshun Steel Co., Ltd., Wuhan, China) plates with a 45° bevel and a 4 mm root face, shown in Figure 1a. The welding process employed was Gas Metal Arc Welding (GMAW), utilizing H08MnSiA welding wire(Nangong Jingkun Welding Materials Sales Co., Ltd., Xingtai, China) as the filler material. A V-groove multi-pass welding technique was applied, consisting of three layers and six passes, arranged in an inverted triangular pattern within the V-groove. The welding process began with the first-layer first pass, measuring 240 mm in length. Each subsequent pass decreased in length by 40 mm. Specifically, the second pass and third pass of the second layer measured 200 mm and 160 mm, respectively, while the sixth pass of the third layer measured 40 mm.

The welded material, with multi-pass weld beads, was cut using wire electrical discharge machining (EDM). The initial cutting position was 15 mm from the edge of the base material, with additional cuts made at 40 mm intervals, resulting in six cuts to obtain cross-sections from the first pass of the first layer to the sixth pass of the third layer. The cutting distance for the EDM was 10 mm, shown in Figure 1b.

After cutting, the samples were successively polished with sandpapers of 800, 1200, 1500, and 2000 grit. Upon completion of the sanding process, the samples were polished using a polishing machine(Changsha Miqi instrument equipment Co., Ltd., Changsha, China) equipped with a silk velvet polishing cloth. The polishing cloth was sprayed with W2.5 diamond suspension for polishing.

The samples were then etched with aqua regia for 7 s and immediately rinsed with water. Aqua regia was prepared by mixing hydrochloric acid (37% by mass) and nitric acid (65% by mass) in a 3:1 volume ratio, with the etching process conducted at room temperature. The prepared samples were observed under a Leica high-power microscope (Leica, Wetzlar, Germany) at a magnification of 16×.

### 2.2. Features of Weld Bead Cross-Section Morphology

**Figure 2:**
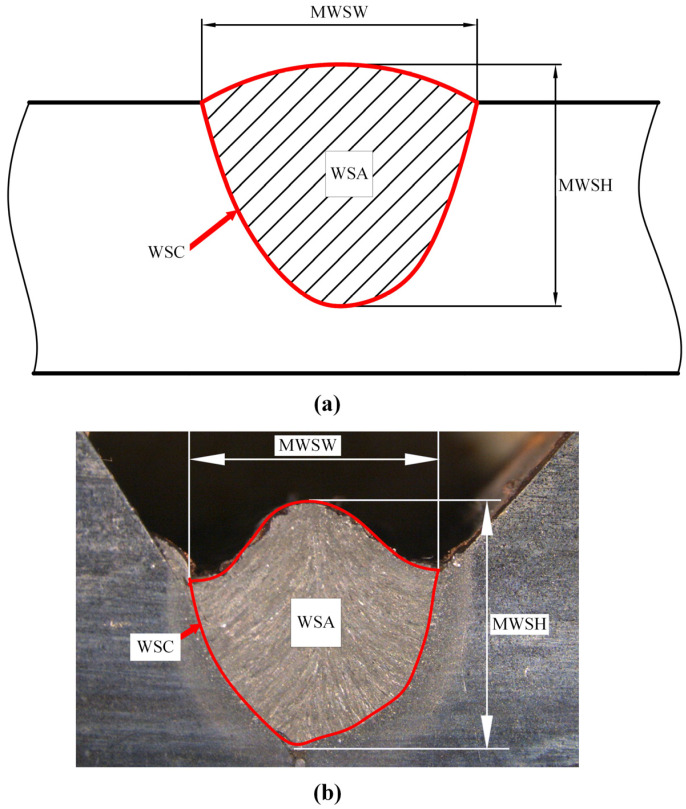
Schematic diagram of weld bead cross-section morphology. (**a**) Schematic diagram. (**b**) Physical schematic diagram.

The weld bead cross-section morphology refers to the cross-section of the weld joint after completion. This process involves dissecting the welded joint, followed by using etching techniques to enhance the clarity of the weld bead’s structure, making it easier to observe and analyze the internal features of the weld bead [17]. Weld bead morphology is observed visually, primarily comprising geometric parameters of the appearance such as weld seam area (WSA), weld seam circumference (WSC), maximum weld seam height (MWSH) and maximum weld seam width (MWSW) as illustrated in Figure 2. Due to processes such as high-temperature heat transfer, melting and solidification, and metal phase transformation involved in welding [9,18], there exists a highly nonlinear relationship between welding process parameters and weld bead profiles. The characteristics of weld bead cross-section morphology are related to many factors; for instance, significant differences exist in weld bead cross-section morphology under different welding speed conditions [19]. And the other factors include the welding current and voltage, different welding methods, weld height, material properties, etc.

### 2.3. Grayscale Segmentation Method for Weld Bead Sectional Images

**Figure 3:**
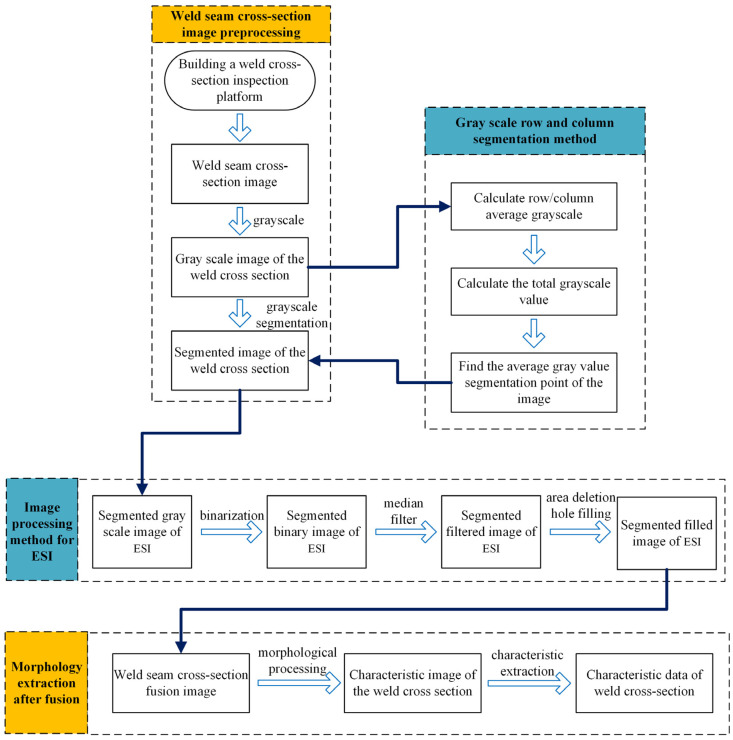
Flowchart of the grayscale segmentation method.

Shown in Figure 3, the technical approach of this paper involves establishing a welding seam detection platform, followed by obtaining the original images of the welding bead sections. These images are then grayscale-converted and subjected to row–column grayscale segmentation. Specifically, the segmentation method calculates the average grayscale value for each row and column in the grayscale image. The total grayscale value of the image is computed, and then, through iteration, the point of segmentation along a row/column is found where the total grayscale values of the upper/lower or left/right halves are equal. This point serves as the boundary for segmentation. After segmentation, the ESI is separately subjected to binarization and median filtering to retain only the main weld bead cross-section morphology. The segmented images are then recombined, and morphological processing is applied to remove irregularities along the edges. Finally, feature extraction is performed on the processed images to obtain welding bead characteristic data.

### 2.4. Gray Scale Analysis of a Weld Bead Cross-Section Image

Images of regions with similar grain structures often exhibit comparable grayscale characteristics. The grayscale values corresponding to the base material and the weld area typically fall within different ranges. Therefore, we can utilize pixel-based grayscale values to process images, extracting the grayscale range corresponding to the weld area and separating it from the base material to obtain the weld bead’s contour.

The issue of uneven grayscale distribution in weld bead images presents a significant challenge in the field of welding image processing. This uneven grayscale distribution often stems from the instability of the light source at the welding site and variations in illumination conditions, thereby directly impacting the visual characteristics of weld bead images. This phenomenon presents formidable challenges for traditional image processing algorithms in the recognition of overall weld beads.

**Figure 4:**
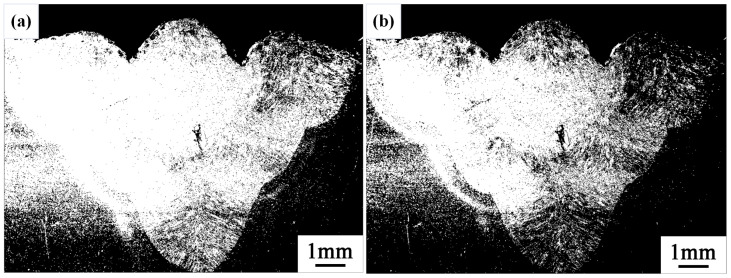
The uneven phenomena during the binarization process. (**a**) Threshold of 75. (**b**) Threshold of 90.

When directly processing the weld bead cross-section morphology of the third-layer sixth pass, differences in grayscale values are observed in the images, shown in Figure 4. Consequently, this leads to uneven phenomena during the binarization process.

**Figure 5:**
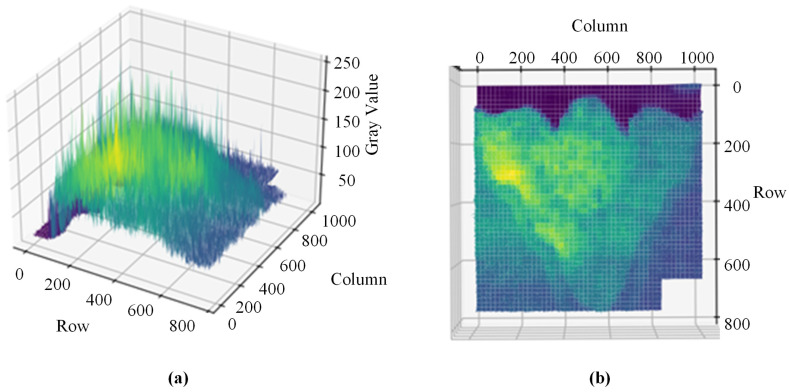
The 3D grayscale image of the weld bead. (**a**) Axial view. (**b**) Top view.

As shown in Figure 5, after analyzing the 3D grayscale image of the weld, a noticeable gradient in grayscale values is observed. Specialized processing is required based on these characteristics to address the uneven grayscale distribution in certain regions, aiming to achieve improved results.

An innovative row–column-based grayscale image segmentation method has been employed to address the challenge of uneven grayscale distribution in weld bead images. Through this approach, local optimization of the weld bead at a microscopic level is achieved in the image. Partitioning along the rows and columns of the image enables the intricate capture of local features in the weld bead image, allowing selective adjustment of areas with uneven brightness. This approach significantly enhances the accuracy of subsequent processing.

### 2.5. Principle of Row and Column Average Gray Value Segmentation

When performing segmentation on grayscale images, there are two approaches: one is based on the peaks of the row–column grayscale image, and the other is based on the average values of the left and right sides of the row–column grayscale image. The advantage of using peak values for segmentation lies in its ability to sensitively capture prominent edges or features in the image, as peaks often correspond to significant changes or boundaries of the target in the image. By identifying peaks in the grayscale image, the image can be segmented into different regions, where the grayscale values change more prominently within each region. However, this method may exhibit excessive sensitivity when dealing with regions where the brightness gradient is relatively smooth. It can be prone to noise interference, leading to unstable segmentation results.

Compared to the peak-based method, the segmentation approach based on average values is more stable and robust. This method calculates the average grayscale value for each row or column and determines the segmentation point based on these averages. This approach is relatively smooth, making it better suited for regions with gradual changes in grayscale values. It is also less susceptible to noise interference. Additionally, the calculation of the average values is relatively simple, leading to higher computational efficiency.

According to the principle of segmentation based on column-wise average grayscale values, the formula is as follows:

(1) $$H=\left[\begin{array}{ccc} h_{11} & \ldots & h_{1n}\\ \vdots & \ddots & \vdots \\ h_{m1} & \cdots & h_{mn} \end{array}\right]$$

(2) $$N_{j}=\frac{\sum_{i=1}^{m}h_{ij}}{m}$$

(3) $$N=\left[\begin{array}{ccccc} N_{1} & \cdots & N_{j} & \cdots & N_{n} \end{array}\right]$$

(4) $$S_{N_{j}}=\sum_{k=1}^{j}N_{k}$$

(5) $$S_{N_{n}}=\sum_{k=1}^{n}N_{k}$$

(6) $$S_{N_{j}}\geq \frac{1}{2}S_{N_{n}}$$

where $h_{ij}$ represents the grayscale value of the individual pixel at the *i*-th row and *j*-th column, H denotes the set of grayscale values for an image with length m and width n, $N_{j}$ is the average grayscale value for the *j*-th column, N represents the set of average grayscale values for each column, and $S_{N_{j}}$ is the sum of grayscale values from the 1-st column to the *j*-th column. In the process of determining the segmentation point, a traversal of the *j* values is conducted. The segmentation column, denoted as $\bar{j}$, is identified as the first *j* value that satisfies the inequality $S_{N_{j}}$ which is greater than or equal to the total grayscale value of the image, $S_{N_{n}}$.

Similarly, based on the principle of segmentation using row-wise average grayscale values, the formula is as follows:

(7) $$M_{i}=\frac{\sum_{j=1}^{n}h_{ij}}{n}$$

(8) $$M=\left[\begin{array}{c} M_{1}\\ \vdots \\ M_{i}\\ \vdots \\ M_{m} \end{array}\right]$$

(9) $$S_{M_{i}}=\sum_{k=1}^{i}M_{k}$$

(10) $$S_{M_{m}}=\sum_{k=1}^{m}M_{k}$$

(11) $$S_{M_{i}}\geq \frac{1}{2}S_{M_{m}}$$

In this case, in the process of determining the segmentation point, a traversal of the *i* values is conducted. The segmentation row, denoted as $\bar{i}$, is identified as the first *i* value that satisfies the inequality $S_{M_{i}}$ which is greater than or equal to the total grayscale value of the image, $S_{M_{m}}$.

### 2.6. Weld Bead Cross-Section Image Processing Methods

#### 2.6.1. Basic Image Processing Methods for Weld Bead Cross-Sections

During the process of capturing weld bead cross-section morphology images, environmental factors and the selection of equipment and facilities may introduce defects in the acquired images. Furthermore, the errors in these images can accumulate and propagate. Therefore, preprocessing of the images is essential to filter out irrelevant noise and interference while retaining the essential information. Dealing with color images can increase the complexity of algorithms. Hence, grayscale images are often used before image processing, or the color images are converted to grayscale. The original weld bead cross-section morphology images inherently possess complexity due to their internal crystal structure and the influence of the cutting profile. To extract contours from these images, it is necessary to process and fill the internal regions. Therefore, thresholding, median filtering, region deletion, and hole-filling operations are applied to achieve effective preprocessing.

1. **Binarize the image after row and column grayscale segmentation**

Fixed thresholding algorithms require manual thresholding, although this process can be time consuming. However, when dealing with illustrations with some complexity and small differences between the noise and the target pattern, a more accurate processing of the pattern can be achieved by manually adjusting the fixed threshold for binarization operations. This fine manual adjustment process helps to cope with subtle differences in complex images and suppresses noise while preserving the target features, thus improving the accuracy of pattern binarization.

2. **Binary image median filtering**

Median filtering is a nonlinear filtering method that is effective in filtering isolated point type noise. The basic principle is to set the pixel value at the anchor point to the median of the grayscale values in the core window centered on the anchor point [20].

3. **Obtain the maximum contour of the filtered image**

The shape of the weld cut surface has many irregular crystal features. Even after the completion of the binarization and median filtering process, there will still be many small regions with scattered distribution, which will bring a series of subsequent detection of many difficulties; thus, the image has been completed to remove most of the noise for the contour detection, and it will be less than the set threshold for the removal of the region which will leave only the largest contour.

4. **Fill the contour image to obtain the weld profile image**

The resulting maximum contour is found by drawing it in a new image and filling in the inner regions to obtain a complete, edge-continuous and noise-free contour of the weld bead.

#### 2.6.2. Edge Detection and Feature Extraction of Weld Bead Cross-Section Image

In the former step, the region containing the weld bead cross-section morphology was identified, and its contour was subsequently detected. Consequently, edge detection and feature extraction can be conducted on this contour to facilitate quantitative analysis of the weld bead cross-section morphology. The pixel values of this largest contour are analyzed to identify the leftmost and rightmost pixel points, thereby determining its MWSW, along with the topmost and bottommost pixel points to ascertain its MWSH.

## 3. Results

### 3.1. Preparation for Sectional Image Row–Column Gray Segmentation

#### 3.1.1. The Original Weld Bead Image Data

**Figure 6:**
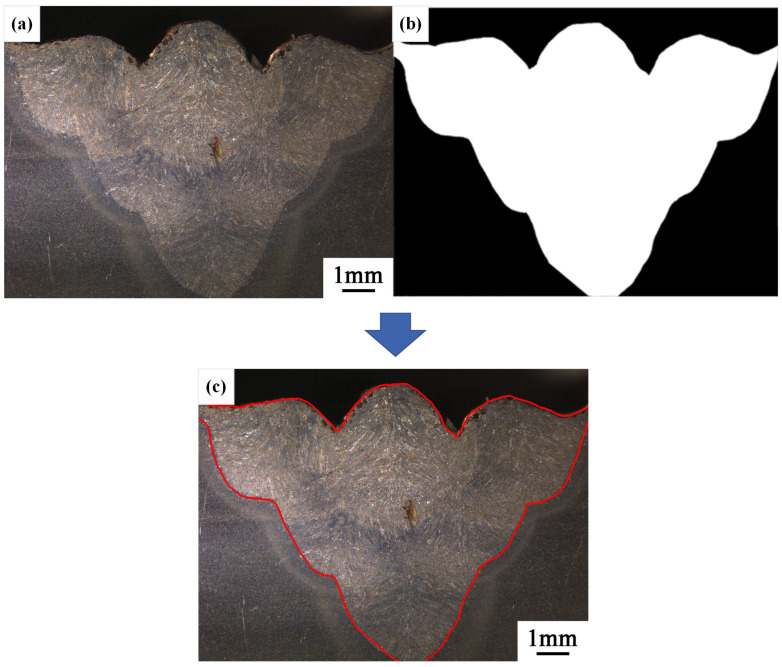
The sample of the third-layer sixth-pass weld bead cross-section image. (**a**) The original image. (**b**) The manual contour. (**c**) The original image overlaid with manual contour.

Before conducting threshold analysis on the segmented image, the contour area of the original image is manually selected and contour extraction is performed, as shown in Figure 6. The collected data includes the following:

WSC: 3289, WSA: 404,529.5, MWSH: 728, and MWSW: 1024.

#### 3.1.2. Sectional Image Gray Segmentation Analysis

**Figure 7:**
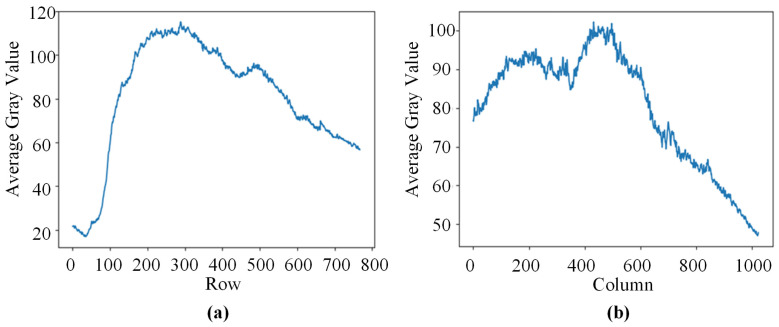
Average grayscale histogram. (**a**) The average gray value of each row. (**b**) The average gray value of each column.

After grayscale conversion of a sample image depicting the third-layer sixth-pass weld bead cross-section morphology, the pixel values were summed and averaged for each row or column. The resulting curves of the row-wise and column-wise average grayscale values are illustrated in Figure 7.

From the average grayscale plot, it is evident that the main feature of the image is brightness in the upper region and darkness in the lower region, with the left side appearing brighter than the right side. It is worth noting that, for the subsequent image, the brightness difference between the left and right sides is even more pronounced.

### 3.2. Gray Average Binarized Image

#### 3.2.1. Binarized Method Threshold Analysis

**Figure 8:**
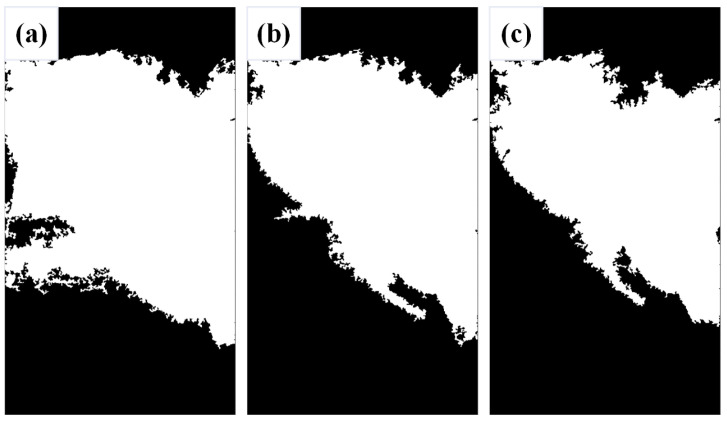
The left half binarized images with different thresholds: (**a**) 90, (**b**) 95, and (**c**) 100.

Following image segmentation, binarization was conducted on the left and right sides of the image, with particular emphasis on delineating the threshold ranges. Initially, a threshold of 90 was set as a baseline for processing the left half of the image. Despite subsequent image processing, certain edge regions remained inadequately identified. Consequently, the threshold was incremented; however, raising it to 100 resulted in partial omission of the weld bead cross-section morphology in the upper region. When the threshold value is varied from 90 to 100, the threshold value 95 was identified as the optimal binarization threshold, yielding satisfactory binarization efficacy for the left half of the image. The difference of these thresholds is shown in Figure 8.

**Figure 9:**
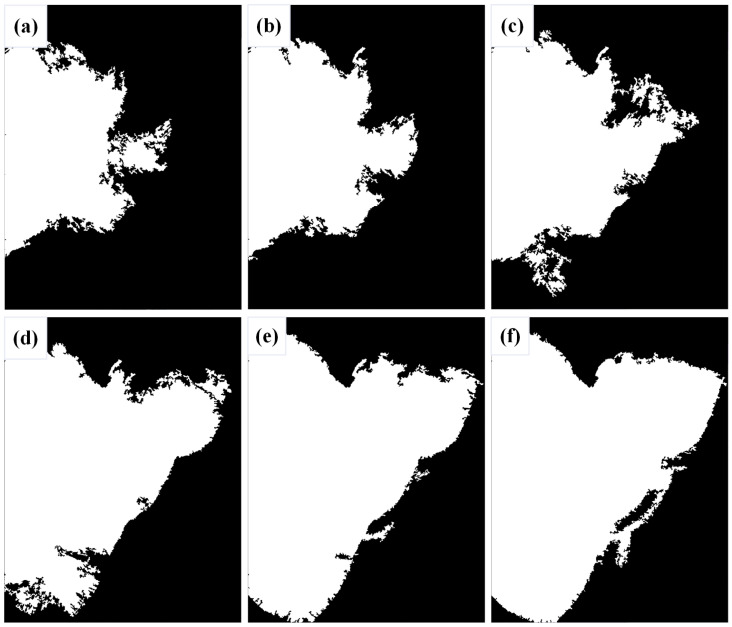
The right half binarized images with different thresholds: (**a**) 90, (**b**) 85, (**c**) 80, (**d**) 75, (**e**) 70, and (**f**) 65.

When addressing the right half of the image, an initial threshold of 90 was employed as a baseline, revealing numerous omitted details. Subsequently, lowering the threshold became imperative to enhance image processing outcomes. By systematically reducing the threshold in increments of five, the threshold that aligned with optimal image processing requisites is progressively identified. The difference of these thresholds is shown in Figure 9.

When the threshold value was varied from 90 to 65, the threshold value 65 was observed when the weld bead cross-section morphology of the right half of the image was well-displayed. Consequently, this threshold value was selected as the final processing result for the right half.

#### 3.2.2. Fusion of Left and Right Profile Images

**Figure 10:**
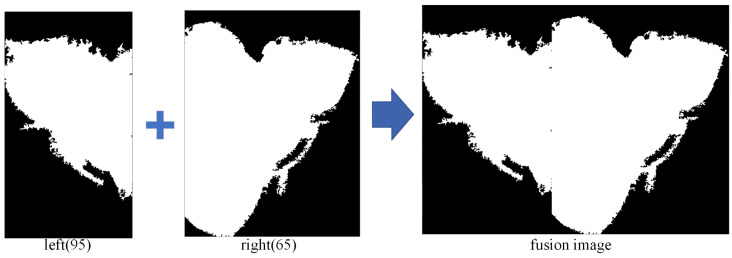
Fusion of left and right profile images.

The results of processing the left and right sides are fused together, with a comparison made between the individually processed images to determine the one that best displays the weld bead cross-section morphology along with its corresponding threshold. Specifically, the threshold values selected were 95 for the left half image and 65 for the right half image, as shown in Figure 10.

### 3.3. Grayscale Averaging Quadripartite Image

#### 3.3.1. Quadripartite Method Threshold Analysis

To address the issue of significant errors observed in the final image compared to the original image in the binary segmentation method, the process is further refined based on average grayscale segmentation. In this step, the image is divided again, this time based on both row and column average grayscale values. The resulting segmented image is then divided into four parts: top-left, top-right, bottom-left, and bottom-right.

**Figure 11:**
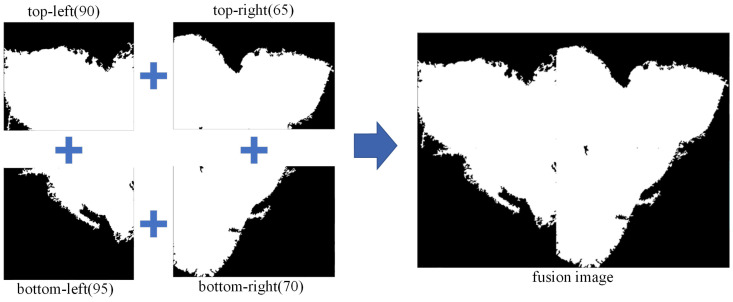
Fusion of the quadripartite profile images.

After image segmentation, binary processing is applied to the quadripartite images. The key is to determine the threshold range for each quadripartite image. By varying the binary threshold every 5 based on the original image, it is observed that a threshold of 90 maximally preserves the contour of the weld bead cross-section. Consequently, the binary threshold for the top-left quadripartite image is set to 90. Similarly, for the top-right, bottom-left, and bottom-right quadripartite images, the binary thresholds are set to 65, 95, and 70, respectively. The overall threshold sequence is (90, 65, 95, 70). This choice aligns with the characteristics of the image: brighter on top, darker on the bottom, brighter on the left, and darker on the right. The fusion image after processing the four quadripartite images is shown in Figure 11.

#### 3.3.2. Morphological Threshold Analysis

**Figure 12:**
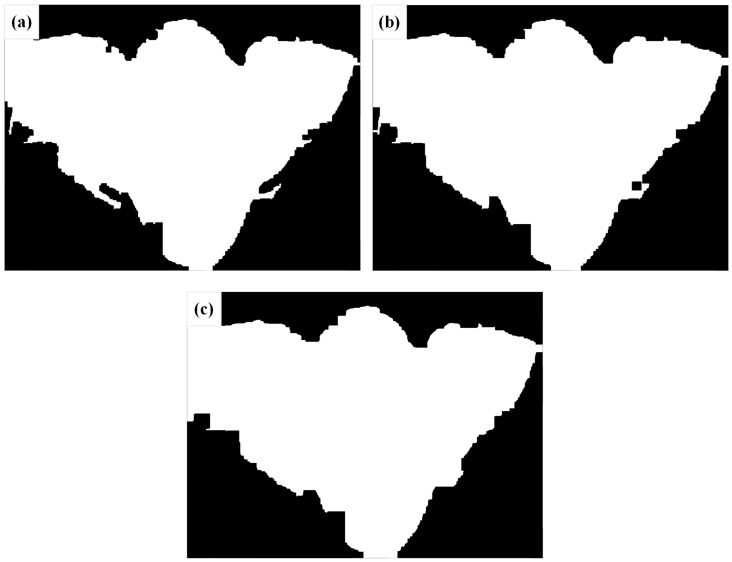
The morphological images with different thresholds: (**a**) (15, 15), (**b**) (25, 25), and (**c**) (35, 35).

After merging the results of processing the four quadripartite images, further morphological processing is required to extract better contours of the image. The obtained morphology contours also depend on the morphological threshold. Here, three threshold values were experimented with, (15, 15), (25, 25), and (35, 35), as shown in Figure 12. The extracted contour data for ESI are presented in Table 1.

After comparing the data, it was observed that as the threshold value increases, the circumference gets closer to the true value, but the area becomes larger, deviating from the true value. However, the difference of MWSH and difference of MWSW remain consistent and are not affected. The relative errors of WSA are 26.8%, 15.1%, and 9.6%, respectively, while the relative errors of WSC are 4.3%, 5.9%, and 6.9%, respectively. The difference of MWSH and difference of MWSW show minimal errors.

## 4. Discussion

### 4.1. Repeatability Tests

#### 4.1.1. Repeatability Tests of the Binary Segmentation Method for Multi-Layer Multi-Pass Weld Bead Sectional Images

**Figure 13:**
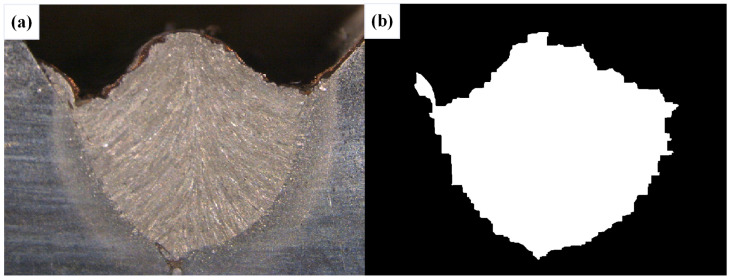
The result of the binary method for the first-layer first-pass image. (**a**) Original image. (**b**) Resulting image.

**Figure 14:**
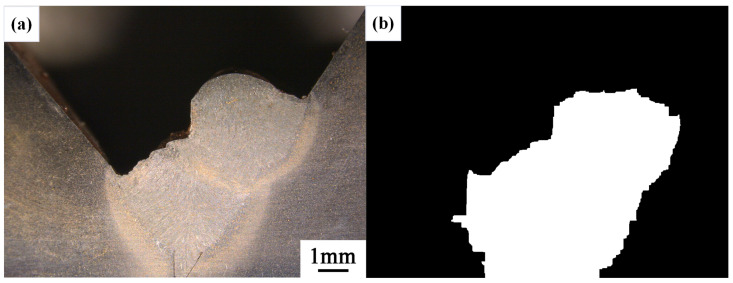
The result of the binary method for the second-layer second-pass image. (**a**) Original image. (**b**) Resulting image.

**Figure 15:**
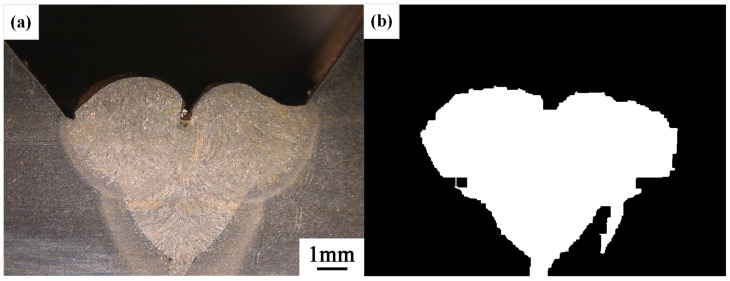
The result of the binary method for the second-layer third-pass image. (**a**) Original image. (**b**) Resulting image.

**Figure 16:**
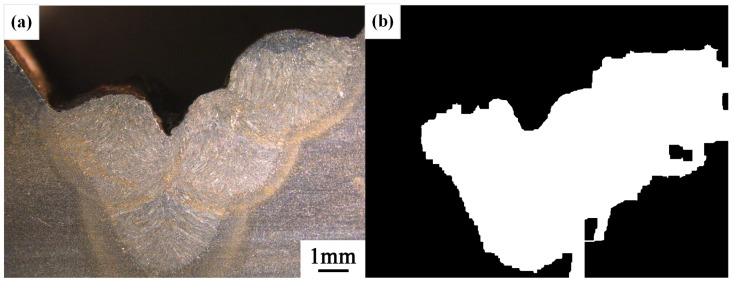
The result of the binary method for the third-layer forth-pass image. (**a**) Original image. (**b**) Resulting image.

**Figure 17:**
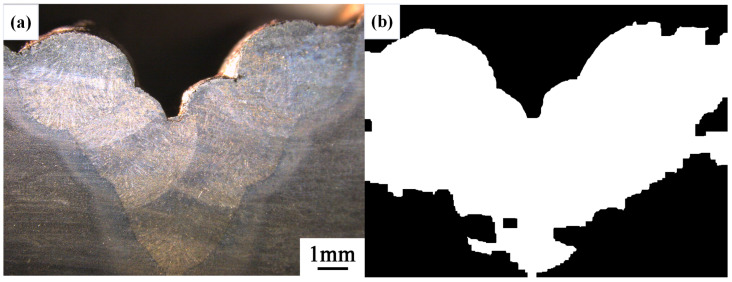
The result of the binary method for the third-layer fifth-pass image. (**a**) Original image. (**b**) Resulting image.

**Figure 18:**
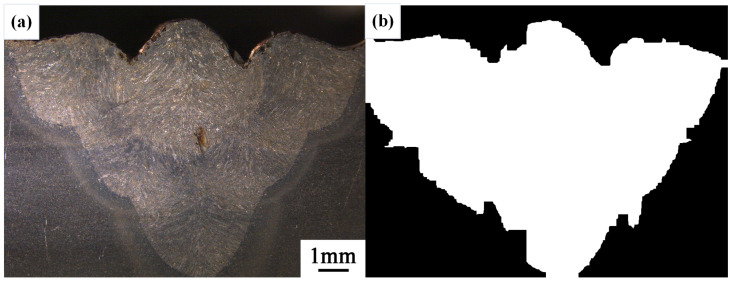
The result of the binary method for the third-layer sixth-pass image. (**a**) Original image. (**b**) Resulting image.

After conducting inspections on the third-layer sixth-pass weld bead images, to validate the method’s applicability, repeatability tests were also performed on weld bead images ranging from the first-layer first-pass to the third-layer fifth-pass configurations, as shown in Figure 13, Figure 14, Figure 15, Figure 16, Figure 17 and Figure 18. Considering the uneven brightness characteristics of each weld bead image, grayscale segmentation was carried out either horizontally or vertically. Subsequently, the binary threshold was gradually adjusted to achieve the closest possible match with the desired morphology contours from the original images.

It can be observed that for the weld bead cross-section morphology of the first-layer first-pass configuration, vertical segmentation was employed with binary threshold values of 132 for the upper part and 125 for the lower part. For the weld bead cross-section morphology of the second-layer second-pass configuration, horizontal segmentation was applied with binary threshold values of 150 for the left part and 135 for the right part. The weld bead cross-section morphology of the second-layer third-pass configuration utilized vertical segmentation with binary threshold values of 120 for the upper part and 150 for the lower part. In the case of the weld bead cross-section morphology of the third-layer fourth-pass configuration, horizontal segmentation was employed with binary threshold values of 120 for the left part and 150 for the right part. For the weld bead cross-section morphology of the third-layer fifth-pass configuration, vertical segmentation was applied with binary threshold values of 125 for the upper part and 100 for the lower part. Lastly, the weld bead cross-section morphology of the third-layer sixth-pass configuration utilized horizontal segmentation with binary threshold values of 95 for the left part and 65 for the right part.

It can be noted that different types of weld bead morphologies require distinct segmentation methods and varying binary threshold values. Thus, automated simplification of the entire process remains challenging, indicating the need for further optimization in the future.

#### 4.1.2. Repeatability Tests of Quadripartite Segmentation Method for Multi-Layer Multi-Pass Weld Bead Sectional Images

**Figure 19:**
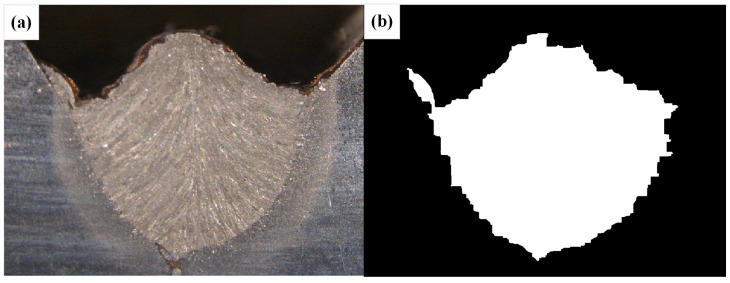
The result of the quadripartite method for the first-layer first-pass image. (**a**) Original image. (**b**) Resulting image.

**Figure 20:**
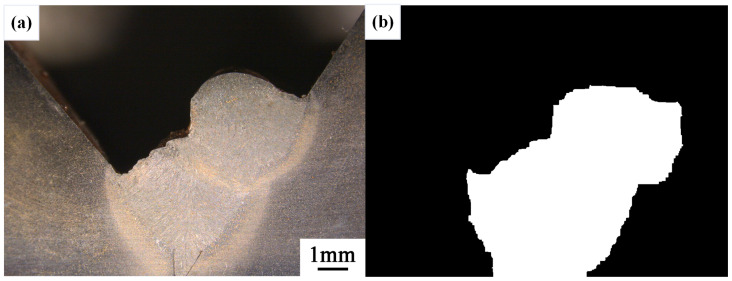
The result of the quadripartite method for the second-layer second-pass image. (**a**) Original image. (**b**) Resulting image.

**Figure 21:**
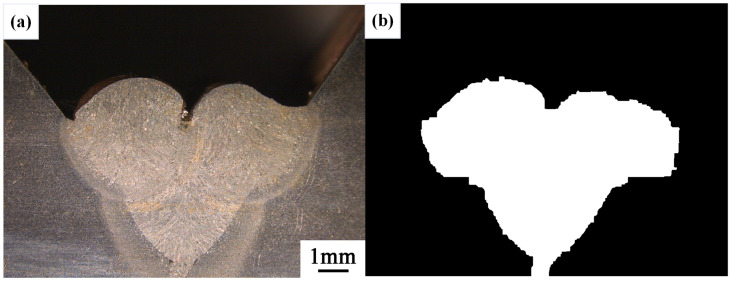
The result of the quadripartite method for the second-layer third-pass image. (**a**) Original image. (**b**) Resulting image.

**Figure 22:**
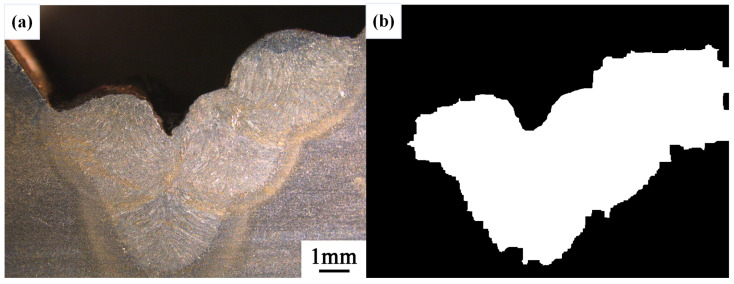
The result of the quadripartite method for the third-layer forth-pass image. (**a**) Original image. (**b**) Resulting image.

**Figure 23:**
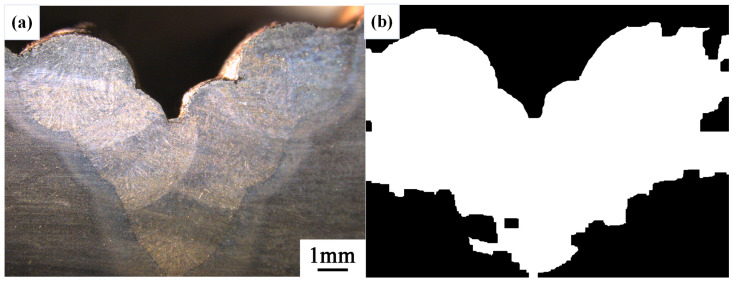
The result of the quadripartite method for the third-layer fifth-pass image. (**a**) Original image. (**b**) Resulting image.

**Figure 24:**
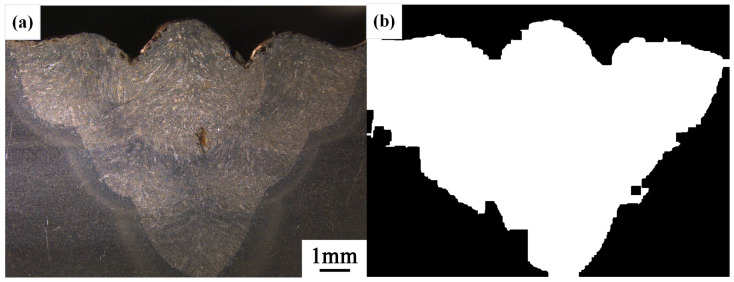
The result of the quadripartite method for the third-layer sixth-pass image. (**a**) Original image. (**b**) Resulting image.

After validating the method’s universality on the third-layer sixth pass of weld bead images, repeatability tests were conducted on weld bead images ranging from the first-layer first pass to the third-layer fifth pass, as shown in Figure 19, Figure 20, Figure 21, Figure 22, Figure 23 and Figure 24. Each weld bead image’s brightness was segmented into four parts. Subsequently, the binary threshold values were gradually adjusted to closely match the required morphological contours in the original images.

It can be observed that for weld bead morphologies with the first-layer first pass, the binary threshold values are (130, 132, 125, 125). For weld bead morphologies with the second-layer second pass, the binary threshold values are (150, 125, 165, 150). For weld bead morphologies with the second-layer third pass, the binary threshold values are (110, 120, 155, 160). For weld bead morphologies with the third-layer fourth pass, the binary threshold values are (110, 150, 130, 160). For weld bead morphologies with the third-layer fifth pass, the binary threshold values are (120, 130, 100, 95). For weld bead morphologies with the third-layer sixth pass, the binary threshold values are (90, 65, 95, 70).

#### 4.1.3. Comparison of Weld Contour Results

**Figure 25:**
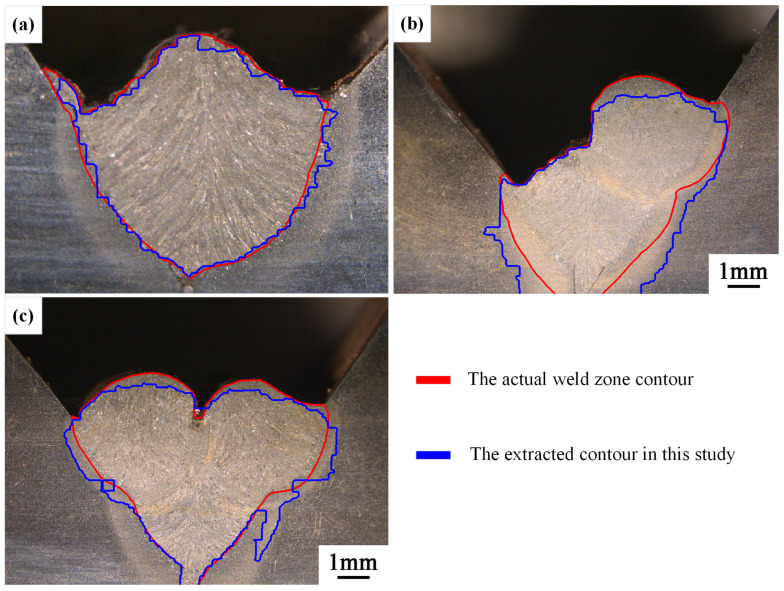
Overlay image of the actual weld zone contour and the contour extracted using the binary method. (**a**) 1st-layer 1st-pass, (**b**) 2nd-layer 2nd-pass, and (**c**) 2nd-layer 3rd-pass.

**Figure 26:**
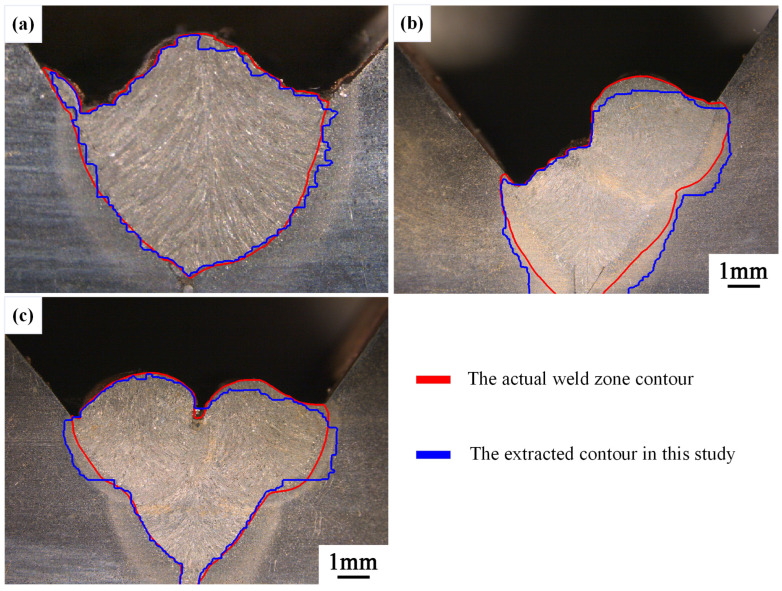
Overlay image of the actual weld zone contour and the contour extracted using the quadripartite method. (**a**) 1st-layer 1st-pass, (**b**) 2nd-layer 2nd-pass, and (**c**) 2nd-layer 3rd-pass.

After extracting the weld morphology contours from the first-layer first pass to the third-layer sixth pass, we selected the welds with higher extraction accuracy (from the first-layer first pass to the second-layer third pass) for contour overlay analysis. The actual weld contour was overlaid with the contours extracted in this study on the original images. Figure 25 shows the results obtained using the binary method, while Figure 26 presents the results obtained using the quadripartite method. It can be observed that the contours extracted using the quadripartite method are generally closer to the actual contours and more accurate compared to the binary method.

The method proposed in this study demonstrates a high degree of contour fitting in certain regions, though some areas exhibit deviations that still align with the overall contour trend. Overall, while the method has not yet achieved precise delineation of the weld contour, it is capable of accurately identifying the general morphology of the weld. This is a new exploration in the field of weld detection.

### 4.2. Analysis and Discussion

#### 4.2.1. Comparison Analysis of Weld Bead Cross-Sectional Feature Data between the Binary Segmentation Method and the Quadripartite Segmentation Method

Following the completion of average grayscale segmentation and the selection of a morphological threshold of 25 for the final image in weld bead cross-section segmentation, the extracted feature data were compared with the original image to compute the errors. Table 2 and Table 3 below present the original feature data for multi-layer multi-pass weld bead cross-sections and the feature data after the binary segmentation process.

Similar to the binary segmentation of weld cross-sections, a comparative analysis of weld cross-sectional feature data is conducted for the quadripartite segmentation method. The obtained data are in Table 4.

**Figure 27:**
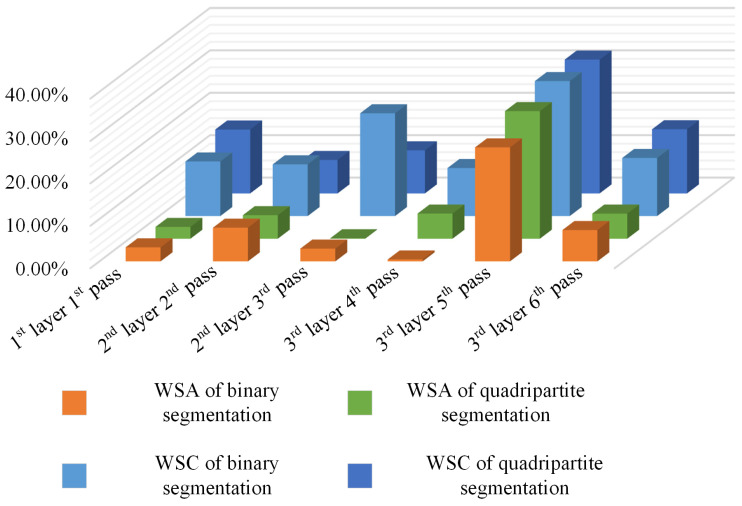
Relative error of the WSC and WSA.

Due to the observation that errors in the MWSH and MWSW are generally small, our primary focus is on errors in terms of WSC and WSA. The relative error is defined as the ratio of the absolute error of the contour data extracted using the method of this paper to the contour data of the original images. The relative errors of the multi-layer multi-pass images after the binary or quadripartite segmentation process are shown in Table 5 and Table 6. We can see a visual comparison in Figure 27.

#### 4.2.2. Comparison Analysis of the Quality of Weld Bead Cross-Sectional Images between the Binary Segmentation Method and the Quadripartite Segmentation Method

**Figure 28:**
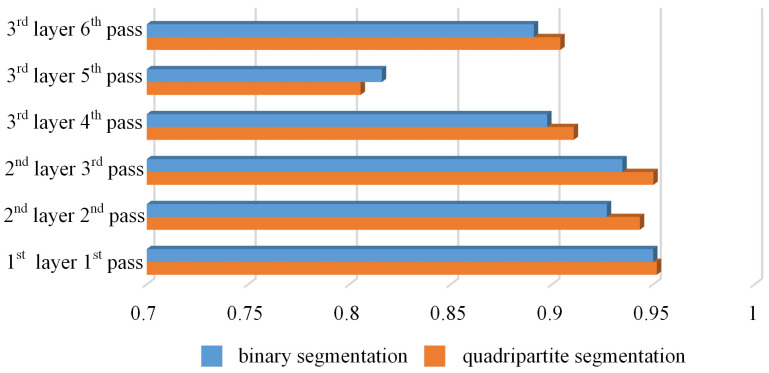
Comparison of SSIM.

**Figure 29:**
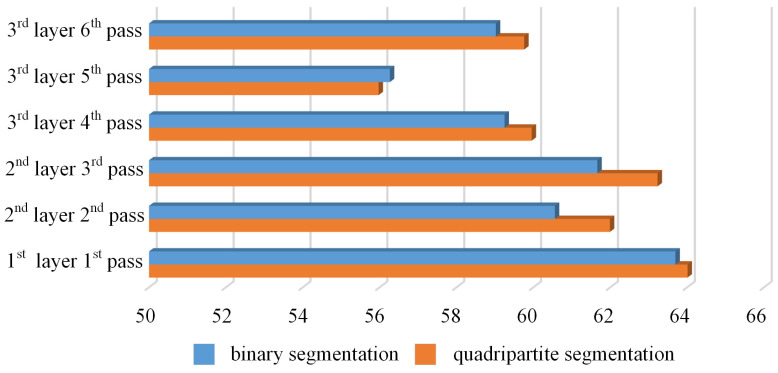
Comparison of PSNR.

Furthermore, evaluations were conducted using the Structural Similarity Index (SSIM) and Peak Signal-to-Noise Ratio (PSNR) for the images generated by both binary and quadripartite segmentation methods, in addition to comparing parameters such as circumference and area of the weld bead cross-section morphology. As shown in Figure 28 and Figure 29, the results indicate that, except for the case of the third-layer fifth-pass weld bead cross-section morphology where the binary segmentation method showed slightly better performance, the quadripartite segmentation method exhibited higher SSIM and larger PSNR in other scenarios. Overall, the quadripartite segmentation method demonstrates superior performance in handling welding bead section images compared to the binary segmentation method.

This row–column-based grayscale image segmentation method provides an effective means to finely process weld bead images based on their local characteristics. Dividing the weld bead image into smaller regions can make more precise brightness adjustments for ESI, thus maximizing the preservation of weld bead details. This not only helps improve the overall visual quality of the weld bead image but also provides a more reliable foundation for subsequent image processing tasks.

## 5. Conclusions

(1) The innovation is the segmentation of the weld bead cross-section morphology into different modules based on the gray value for image processing. The ESI has an individual threshold, which allows the weld bead cross-section morphology to be extracted by region. The individually extracted morphology is then fused to obtain the complete weld bead cross-section morphology.
(2) A segmentation points extraction method based on the average gray value in the row and column is proposed. This method solves the uneven distribution of the gray value of the weld image after segmentation and can extract a more convenient environment for the subsequent processing.
(3) Multi-layer multi-pass weld bead cross-section morphology extraction experiments were conducted, ranging from first-layer first pass to third-layer sixth pass. The relative errors in WSC and WSA are within 10%, while the discrepancies in MWSW and MWSH can be close to the true value. The quality assessment falls within a reasonable range, which the average value of SSIM is superior to 0.9 on average and the average value of PSNR is superior to 60. This indicates that the multi-layer multi-pass weld feature extraction method based on image processing, while requiring a certain level of image quality, can identify the general contour of the weld bead and extract its shape parameters. We have reason to believe that in future work, more complex methods can be integrated to further enhance the accuracy and precision of the identification process, ultimately replacing manual identification.

## Figures and Tables

**Table 1:** The contour data of the morphological images with different thresholds.

Threshold	Original Figure	(15, 15)	(25, 25)	(35, 35)
WSC (pixel)	3289	4169	3787	3604
WSA (pixel^2^)	404,529.5	422,026.5	428,483.0	432,633.5
MWSH (pixel)	728	726	726	726
MWSW (pixel)	1024	1024	1024	1024

**Table 2:** The contour data of the multi-layer multi-pass original images.

Original Image	1st-Layer 1st-Pass	2nd-Layer 2nd-Pass	2nd-Layer 3rd-Pass	3rd-Layer 4th-Pass	3rd-Layer 5th-Pass	3rd-Layer 6th-Pass
WSC (pixel)	2507.2	2027.4	2224.6	2927.0	3419.6	3289.1
WSA (pixel^2^)	286,721.5	200,675.0	223,407.0	307,748.0	347,121.5	404,529.5
MWSH (pixel)	651	578	565	701	729	728
MWSW (pixel)	761	609	684	902	1024	1024

**Table 3:** The contour data of the multi-layer multi-pass images after the binary segmentation process.

Binary Segmentation	1st-Layer 1st-Pass	2nd-Layer 2nd-Pass	2nd-Layer 3rd-Pass	3rd-Layer 4th-Pass	3rd-Layer 5th-Pass	3rd-Layer 6th-Pass
WSC (pixel)	2829.7	2274.7	2763.7	3258.5	4508.1	3739.7
WSA (pixel^2^)	277,245.0	216,625.0	230,121.0	309,118.5	440,427.0	434,406.5
MWSH (pixel)	643	535	539	657	721	726
MWSW (pixel)	748	649	728	867	1024	1024

**Table 4:** The contour data of the multi-layer multi-pass images after the quadripartite segmentation process.

Quadripartite Segmentation	1st-Layer 1st-Pass	2nd-Layer 2nd-Pass	2nd-Layer 3rd-Pass	3rd-Layer 4th-Pass	3rd-Layer 5th-Pass	3rd-Layer 6th-Pass
WSC (pixel)	2884.9	2187.4	2450.4	3215.2	4498.5	3786.6
WSA (pixel^2^)	278,755.0	211,807.0	223,177.0	289,484.5	451,563.5	428,483.0
MWSH (pixel)	643	543	562	624	719	726
MWSW (pixel)	767	613	731	908	1024	1024

**Table 5:** The relative errors of the multi-layer multi-pass images after the binary segmentation process.

Binary Segmentation Error	1st-Layer 1st-Pass	2nd-Layer 2nd-Pass	2nd-Layer 3rd-Pass	3rd-Layer 4th-Pass	3rd-Layer 5th-Pass	3rd-Layer 6th-Pass
WSC	12.86%	12.20%	24.23%	11.32%	31.83%	13.70%
WSA	3.31%	7.95%	3.01%	0.45%	26.88%	7.39%

**Table 6:** The relative errors of the multi-layer multi-pass images after the quadripartite segmentation process.

Quadripartite Segmentation Error	1st-Layer 1st-Pass	2nd-Layer 2nd-Pass	2nd-Layer 3rd-Pass	3rd-Layer 4th-Pass	3rd-Layer 5th-Pass	3rd-Layer 6th-Pass
WSC	15.07%	7.89%	10.15%	9.85%	31.55%	15.12%
WSA	2.78%	5.55%	0.10%	5.93%	30.09%	5.92%

## Data Availability

The original contributions presented in the study are included in the article, further inquiries can be directed to the corresponding author.
